# ACTH vs betamethasone for the treatment of acute gout in hospitalized patients: A randomized, open label, comparative study

**DOI:** 10.31138/mjr.29.3.178

**Published:** 2018-09-27

**Authors:** Dimitrios Daoussis, Panagiotis Kordas, Collaborators

**Affiliations:** University Hospital of Patras, University of Patras Medical School, Division of Internal Medicine, Department of Rheumatology; Department of Rheumatology, University of Thessaly Medical School, University Hospital of Larissa

**Keywords:** gout, treatment, ACTH, Adrenocorticotropic hormone, hospitalized patients

## Abstract

**Background:**

Hospitalized patients usually have significant comorbidities and receive multiple medications which leads to a high frequency of contraindications to standard treatment options for acute gout (NSAIDs, colchicine, steroids). Adrenocorticotropic hormone (ACTH) has long been used in acute gout, exhibiting significant clinical efficacy and an excellent safety profile.

**Aim:**

To assess 1) the clinical efficacy of ACTH in gout compared to betamethasone in hospitalized patients 2) the safety profile of ACTH vs betamethasone and 3) the effect of ACTH on immune responses and metabolic parameters.

**Methods:**

This is a randomized, open label comparative study directly comparing ACTH vs betamethasone for acute gout. We plan to recruit 60 hospitalized patients who will be randomly assigned to either the ACTH or the betamethasone group on a 1:1 basis. Patients will be clinically assessed at baseline and at 24, 48, 72h and 5days time points. (Intensity of pain, physician and patient global assessment, tenderness, swelling and redness). Serum and plasma samples will be collected at baseline and at the 24, 48, 72h time points from all study subjects. We will assess the effect of ACTH vs betamethasone on several metabolic parameters concentrating on glucose homeostasis.

**Results:**

The study is currently recruiting patients.

**Conclusions:**

If the efficacy and safety profile of ACTH is verified in this randomized controlled trial, the use of ACTH for the treatment of gout in the hospital setting will be strongly supported.

## BACKGROUND

Treatment of gout can be either simple and straightforward in many cases, or extremely problematic in others. Current guidelines recommend the use of non-steroidal anti-inflammatory drugs (NSAIDs) and colchicine as first-line treatment.^1^ However, patients with gout typically have multiple comorbidities that often preclude the use of these drugs.^2^ The management of gout can be even more troublesome in the hospital setting. Hospitalized patients usually have significant comorbidities and receive multiple medications, which lead to a high frequency of contraindications to the above agents. Steroids are considered an alternative therapeutic option especially for “difficult-to-treat” patients such as hospitalized patients. However they associate with immunosuppression and metabolic side effects; therefore, their use in the inpatient setting may be problematic as well.^3^

Adrenocorticotropic hormone (ACTH) has long been used in gout. The interest in ACTH was revived in the mid-1990s when several studies showed that ACTH is equally effective and, in most cases, acts faster than NSAIDs and steroids and exhibits an excellent safety profile.^4–6^

It was previously thought that the anti-inflammatory action of ACTH was steroid related: if this was the case, then one could argue that treatment with ACTH would have no advantage over the systemic administration of steroids. However, experimental evidence challenges this view; it has been shown that ACTH mainly acts in a steroid independent manner.^7–8^ We have been using ACTH as a first-line treatment for hospitalized patients since 1995. We have recently reported our experience with ACTH in gout in hospitalized patients - we found that it is highly effective and associates with minimal side effects.^9^

## AIM

To assess 1) the clinical efficacy of ACTH in gout compared to betamethasone in hospitalized patients; 2) the safety profile of ACTH vs betamethasone; and 3) the effect of ACTH on immune responses and metabolic parameters.

## HYPOTHESIS

Based on our retrospective data related to the use of ACTH in gout, we hypothesized that ACTH has comparable clinical efficacy to betamethasone in the treatment of acute gout but exhibits a better safety profile and associates with less immunosuppression than betamethasone.

## STUDY DESIGN

We plan to recruit 60 hospitalized patients with acute gout. Patients will be randomly assigned to either the ACTH or the betamethasone group on a 1:1 basis.

All patients will provide written informed consent. The study protocol has already been submitted and approved by the Ethics Committee of the University Hospital of Patras.

The following data will be recorded for each patient: 1) age; 2) gender; 3) admission and discharge diagnosis; 4) history of major comorbidities that represent contraindications to established gout therapies; and 5) history of hyperuricemia and gout. Comorbidities that will be recorded: a) hypertension defined as having a blood pressure (BP) of ≥140mmHg systolic and/or ≥90 mmHg diastolic and/or receiving any antihypertensive therapy; b) cardiovascular disease defined as the presence of coronary heart disease, cerebrovascular accident or peripheral vascular disease; c) chronic kidney disease defined as an estimated glomerular filtration rate, measured by the abbreviated Modification of Diet in Renal Disease formula of 60–90ml/min/1.73m^2^ (mild) or <60ml/min/1.73m^2^ (moderate/severe); and d) diabetes mellitus (DM) defined as fasting serum glucose levels >126mg/dl and/or the use of oral hypoglycemic medications or insulin.

Patients will receive an IM injection of either 100 IU of ACTH (Synachten Depot) or 6 mg of betamethasone (Celestone Chronodose, which is the most commonly used intramuscular steroid formulation in our country - frequently used for the treatment of gout in the hospital setting). In case of partial or no response, patients will receive a second IM injection of the same drug at the 24h time point.

Patients will be assessed at baseline and at 24, 48, 72h and 5-day time points. The clinical efficacy will be assessed as follows:

1. Intensity of pain will be recorded using a Visual Analogue Scale (0–10 cm) at 24, 48, 72h and 5days time points. Pain VAS will be also recorded at the 6 and 12h time points (self reported by the patient on a special diary provided);
2. Physician and patient global assessment (0–100 scale) at 24, 48, 72h and 5days time points;
3. Tenderness, swelling and redness (0–3 scale) will be recorded at 24, 48, 72h and 5days time points.

## LABORATORY ANALYSIS

We aim to assess the effects of ACTH vs betamethasone on i) metabolic parameters and ii) immune responses.

### i) Metabolic parameters

Serum and plasma samples will be collected at baseline and at the 24, 48, 72h time points from all study subjects and will be stored at −70°C. We will assess the effect of ACTH vs betamethasone on several metabolic parameters concentrating on glucose homeostasis.

We propose the measurement of the following molecules in serum samples collected at the time points specified above:

1. Fasting glucose, insulin and C-peptide so we can explore the effect of ACTH vs betamethasone on glucose homeostasis;
2. Total cholesterol, LDL, HDL and triglycerides so we can explore the effect of ACTH vs betamethasone on lipid homeostasis;
3. Cortisol levels in patients treated with ACTH. So far, the effect of a single IM ACTH injection of 100IU on cortisol levels is not known. We hypothesize however, that cortisol levels will raise soon following the ACTH injection and decline shortly thereafter, thus causing minimum immunosuppression. In betamethasone treated patients we propose the measurement of both Cortisol (using an assay with no cross reactivity with betamethasone) and ACTH levels. We hypothesize that betamethasone will have a more pronounced effect on the HPA axis than ACTH and therefore may associate with a more intense and long-lasting immunosuppression;
4. Tetracosactide (Synachten) levels and betamethasone levels in ACTH and betamethasone treated patients respectively. Tetracosactide will be measured by ELISA methodology in plasma samples (Elisa Kit from Peninsula Laboratories, San Carlos, USA) that reacts with the part 1–24 of ACTH (and thus Tetracosactide). Plasma samples will be prepared by total elimination of endogenous ACTH by cation exchange chromatography. Similarly, betamethasone levels will be measured by ELISA methodology.
  With these experiments we, will explore how long tetracosactide and betamethasone circulate in peripheral blood following a single intramuscular injection. We will match these data with the data derived from the experiments presented above (cortisol levels) to explore the effect of tetracosactide (Synachten) vs betamethasone on the HPA axis.

### ii) Effects on immune responses

We will study the effects of ACTH vs betamethasone on key cells mediating immune responses, such as a) T cells and b) neutrophils. We propose the study of 10 patients (5 in the ACTH and 5 in the betamethasone group) matched for age and gender. These patients will not have active infection.

#### A) Effects on T cells

We will collect 20 ml of heparinized peripheral blood at baseline and at the 24h time point. Peripheral blood mononuclear cells (PBMC) will be separated using a standard Ficoll centrifugation protocol and will be immediately stored in liquid nitrogen. We estimate to collect 20×10^6^ PBMC’s from each sample.

When all samples have been collected, we will use flow cytometry to assess the expression of several activation markers on T cells. PBMC’s will be stained with fluorochrome-conjugated monoclonal antibodies against CD3, CD40L, CD69 and CD25 and their respective isotypic controls. CD40L, CD69 and CD25 are typical T cell activation markers. Flow cytometry will be used to assess the percentage of CD3^+^ CD40L^+^, CD3^+^ CD69^+^ and CD3^+^ CD25^+^ double positive cells. We also propose to assess these activation markers on T cells following activation. PBMC’s will be cultured in RPMI supplemented with 15% FBS and antibiotics (pen/strep) for 48h. PHA at a concentration of 1μg/ml will be added to culture medium to activate T cells. Following 48h of culture, cells will be collected by centrifugation and analyzed as above using flow cytometry. In this experiment we also plan to measure IL-2 levels in the culture supernatants since IL-2 is the main cytokine produced following T cell activation. With these experiments we will explore the effects of ACTH vs betamethasone on T cells which are key cells mediating adaptive immune responses. We hypothesize that ACTH has less effects on T cells compared to betamethasone. If this hypothesis is confirmed by the experiments presented above, it will provide indirect evidence that ACTH causes less immunosuppression than steroids.

## SIGNIFICANCE OF THE STUDY

This will be the first study to assess the efficacy of ACTH in gout in the hospital setting in a prospective manner. We have previously reported that ACTH is highly effective in these patients and exhibits an excellent safety profile; however, our data were retrospectively collected. Therefore, if the efficacy and safety profile of ACTH is verified in this randomized controlled trial, the use of ACTH for the treatment of gout in the hospital setting will be strongly supported. However, we believe that the laboratory part of the study will provide even more valuable data. If ACTH is proven to cause less metabolic abnormalities and associates with less immunosuppression compared to betamethasone, then ACTH may become the treatment of choice in this setting. Considering that hospitalized patients are the most difficult-to-treat patients, this may lead to wider use of ACTH in the treatment of gout in the community as well.
